# Highly selective heteroaromatic sulfur containing polyamides for Hg^+2^ environmental remediation

**DOI:** 10.1080/15685551.2020.1727172

**Published:** 2020-02-13

**Authors:** Soha M. Albukhari, Mahmoud A. Hussein, Mona A. Abdel Rahman, Hadi M. Marwani

**Affiliations:** a Department of Chemistry, Faculty of Science, King Abdulaziz University, Jeddah, Saudi Arabia; b Polymer Chemistry Lab. 122, Chemistry Department, Faculty of Science, Assiut University, Assiut, Egypt

**Keywords:** Heteroaromatic sulfur, polyamides, synthesis & characterizations, Hg^+2^, environmental remediation, surface selectivity

## Abstract

Environmental remediation concerns about pollution and contamination removal from environmental media, such as soil, air, or surface water. Enormous efforts have been applied in metal removal from surface water. In this study, four novel heteroaromatic sulfur-containing polyamides **6_a-d_** carry both types of aliphatic and aromatic species in their polymer backbones as selective adsorbents for Hg^+2^ metal ion from aqueous solution have been synthesized in considerable amounts. The polycondensation method at low temperature is used as a simple and low coast polymerization technique. This occurred by the interaction of the thiophene-based monomer **5** with different diacid chlorides of both types. Beforehand the polymerization, the structures of monomer **5** were confirmed by spectral and elemental analyses. Also, the structures of the new polymers were investigated by both spectral and elemental analysis; besides their solubility, GPC data, XRD diffraction patterns, thermal analysis, and FE-SEM micrographs. The synthesized polymers were freely soluble in polar protic solvents due to the presence of heteroaromatic sulfur functional groups. Furthermore, the analytical competition of the new polymers has been tested using inductively coupled plasma-optical emission spectrometry (ICP-OES) for its selective extraction across different metal ions. Polymer **6_c_** was the most selective toward Hg^+2^ and considered as a highly selective adsorbent for Hg^+2^ environmental remediation among all derivatives and its adsorption detection and efficiency were also investigated. Polymer 6c showed the most effective adsorption quantity on its surface at pH = 1. Moreover, the calculated adsorption isotherm showed a typical isotherm to the Langmuir adsorption type. This showed that the adsorption capacity of polymer **6_c_** for Hg^+2^ was 47.95 mg g^−1^. These novel polymers are serving as simple and inexpensive heavy metal ions adsorbent materials from drinking water and wastewater.

## 1. Introduction

In recent years, Environmental Remediation study has become economically and environmentally attractive. Surface water polluted with heavy metal ions has a high impact on the environment. The medical, industrial, household, agricultural, and technological applications spread toxic metals in the environment, which has a negative potential impact and critical disruption on human health and environmental balance [1, 2]. Enormous efforts have been applied to extract metal from water and wastewater streams [3, 4].

Polythiophene, polyaminothiozole, and polyamide are modern types of hybrid linear polymers. These organic-inorganic polymers have high selectivity toward metal removal due to the existence of thiophene and arylidene moieties in the polymer backbones [5, 6]. Polythiophene has great synthetic flexibility with different derivatives that have interesting electrochemical and optical properties. It is widely used as a semiconductor polymer in photovoltaic, sensor, and transistor industries [7, 8]. More particularly, polyamide and polyaminothiozole play an important role in industrial and commercial products due to its photo-curing properties, chemical resistance, electronic conductivity, high thermal stability, high mechanical properties, fire retarding properties, and interesting biological activity in different fields [9, 10]. Chemical modification of polymer with amine groups has a high impact on polymer properties [11]. Amine groups are rapidly protonated in acidic media and form an electrostatic attraction between anionic compounds, including metal anions. The dual functionality of these polymers added both anionic and cationic character to the product, which enhances the removal capacity toward metal anions [12].

Generally, metal removal from contaminated water includes chemical precipitation, evaporation, electrodeposition, nanofiltration, electrodialysis, and reverse osmosis methods [13]. Although these methods are simple and fast but are more efficient in high concentrations only [14]. Other analytical methods are widely used and include inductively coupled plasma-optical emission spectrometry (ICP-OES) [15], ion chromatography [16], atomic absorption spectroscopy [17], anodic stripping voltammetry [18], which consider efficient methods for metal ions removal. However, the contaminated water has various metal ions, which are toxic for the environment, and selectivity of metal ion removal is desired. Therefore, liquid-liquid extraction, ion exchange, and solid-phase extraction can be used as selective metal ion removal methods [19]. Furthermore, biomass polymer materials can be applied to heavy metal removal when it combined with ion exchange or membrane-based separation technique. The sorption capacity and selectivity of metal ion increased depend on chemical modification of the biomass polymer, which considers an eco-friendly method for heavy metal removal [20].

In this approach, four novel heteroaromatic sulfurs-containing polyamides **6_a-d_** carry both types of aliphatic and aromatic species in their polymer backbones as selective adsorbents for Hg^+2^ metal ion from aqueous solution have been synthesized polycondensation method. The new polymers have been identified by elemental as well as spectral analyses. Moreover, the characterizations are studied by GPC, XRD, and FE-SEM techniques. ICP-OES is used to measure the selective extraction toward different metal ions. The significant prospect is observed in Hg^+2^ adsorption. Its adsorption efficiency is the highest among all derivatives and its adsorption detection and efficiency for other metals removal were also investigated. These novel polymers have shown high efficiency in the metal removal from polluted water, as their expansion will open the field for the development of materials with high metal removal efficiency.

## 2. Experimental

### 2.1. Materials and chemicals

Cyclopentanone (BDH, 99%), thiophencarbaldehyde (Fluka, 95%), ethanol (BDH, 99.7–100%), chloroacetylchloride (Merck, 97%), *N*-methylpyrrolidone (Merck, NMP), and anhydrous aluminum chloride (Sigma-Aldrich). All chemicals are used as purchased, without further purifications. More particularly, Adepoyl- and sebacoyl dichlorides (Sigma-Aldrich) are distilled at 125°C/11 Torr and at 182°C/16 Torr, respectively, before used [21]. Terphthaloyl- and isophthaloyl chlorides (Sigma-Aldrich) are purified by crystallization from *n*-hexane (m.p.: 83–84°C and 40°C), respectively. Other reagents such as solutions of 1000 mgL^−1^ Cd(II), Co(II), Cu(II), Cr(III), Fe(III), Ni(II) and Zn(II) are used as stock standard solutions (Sigma-Aldrich, Milwaukee, WI, USA). All other reagents are used in spectral purity grade, and with double-distilled deionized water throughout the experimental studies.

### 2.2. Monomer synthesis

#### 2.2.1. 2,5-bis(Thiophen-2-ylmethylene)cyclopentanone (3)

This pre-monomer was synthesized as the following: A mixture of 2-thiophencarbaldehyde (2.01 mL, 22 mmol) and cyclopentanone (0.84 g, 10 mmol) was dissolved in 50 mL absolute ethanol in existence of 3 mL freshly prepared KOH alcoholic solution [22 23. The mixture was stirred for 8 h at 25°C. A solid participate separated out by filtered and dried off. A yellow precipitate was obtained by recrystallization of the product using methanol. Yield: 80%; m.p.: 146°C. Anal. Calcd. for C_15_H_12_OS_2_: C, 66.14; H, 4.44; S, 23.54. Found: C, 65.95; H, 4.55; S, 23.48. IR (KBr) υ Cm^−1^: at 1641 (s, C=O cyclopentanone), at 1585–1600 (s, C=C, arylidene linkage), at 744 (w, C–S), at 2943 (w, CH stretching of aliphatic), at 3145 (w, CH stretching of aromatic). ^1^H-NMR (CDCl_3_): δ = 3.07 (s, 4H, 2CH_2_ of cyclopentanone), 7.18 (dd, 2H, Ar-H), 7.43 (d, 2H, Ar-H), 7.59 (s, 2H, Ar-H), 7.83 (s, 2H, 2CH=C). ^13^C-NMR (CDCl_3_): δ = 195.09, 140.40, 135.80, 132.67,130.40, 128.14, 126.29, 26.02 which is in agreement with the proposed structure.

#### 2.2.2. 2,5-bis[4-Choloroacetyl(thiophen-2-ylmethylene)]cyclopentanone (4)

This chloroacetylated monomer was synthesized according to the normal condition of Friedel-Crafts reaction as follows: a mixture of chloroacetylchloride (2.26 g, 20 mmol) and bis-thiophene pre-monomer **1** (2.72 g, 10 mmol) was dissolved in 60 ml of carbon disulfide, followed by adding anhydrous aluminum chloride (5.34 g, 40 mmol) in little portions^23^. The reaction mixture was stirred in an ice bath for 6 h. The carbon disulfide was evaporated, and the residue was poured into cold hydrochloric acid. By filtration, the yellow precipitate was collected and washed with excess water, dried, and recrystallized from ethanol. Yield: 83%, m.p.: 167°C. Anal. Calcd. for C_19_H_14_O_3_Cl_2_S_2_: C, 53.64; H, 3.29; Cl, 16.70; S, 15.06. Found: C, 52.86; H, 3.92; Cl, 15.95; S, 14.64. FT-IR (KBr) (cm^−1^): υ = 1593 (s, C=C arylidene linkage), 1638 (s, carbonyl of cyclohexanone), 1681 (s, carbonyl of chloroacetyl), 2957 (m, CH stretching of aliphatic), 3073 (m, CH stretching of aromatic). ^1^H-NMR (CDCl_3_): δ = 1.66 (t, 4H 2CH_2_ cyclopentanone), 3.21 (s, 4H 2CH_2_ chloroacetyl),7.84–7.17 (m, 2H 2CH=C of arylidene and 4H, 2 thiazole moieties). ^13^C-NMR (CDCl_3_): δ = 195.45, 180.65, 148.23, 145.35,141.17, 133.57, 128.64, 44.28, 29.86 which is in agreement with the proposed structure.

#### 2.2.3. 2,5-bis [2- Aminothiazol-4-yl(thiophen-2-ylmethylene)]cyclopentanone (5)

This monomer was synthesized as follows: A mixture of thiourea (0.76 g, 10 mmol) and chloroacetylated monomer 4 (2.125 g, 5 mmol) was dissolved in 25 ml of absolute ethanol and refluxed for 6 h. The obtained solution was poured onto 25 ml of previously prepared 10% cold sodium acetate solution. By filtration, solid precipitate was obtained and dried for 48 h. Brownish crystals were obtained from ethanol, yield 87%, m.p. 283°C. Anal. Calcd. for C_21_H_16_ON_4_S_4_: C, 53.85; H, 3.42; N, 11.96; S, 27.35. Found: C, 54.63; H, 3.87; N, 12.47; S, 26.96. FT-IR (KBr) (cm^−1^): υ = 1580 (s, C,C double bond of thiophenylidene bond), 1635 (s, carbonyl of cyclopentanone moiety), 2967 (m, CH stretching of aliphatic), 3088 (m, CH stretching of aromatic), 3355, 3190 (w, primary amino groups). ^1^H-NMR (CDCl_3_): δ = 2.88 (m, 4H, 2CH_2_ of cyclopentanone moiety), 8.24–7.73 (m, 2H, 2CH=C of thiophenylidene, and 4H, thiophene, 2H, thiazole moieties), 9.46 (s, 4H primary amino groups).

### 2.3. Sulfur-containing polyamides (6_a-d_)

A new series of sulfur-containing polyamides were synthesized as follows: A condenser connected with a three-necked round flask was used under a liquid nitrogen atmosphere. A solution of different diacid chlorides (including: terephthaloyl-, isophthaloyl-, adepoyl-, and sebacoyl-) (2 mmol) was dissolved in 10 mL of NMP and then added in a dropwise to a mixture of monomer **5** (2 mmol) and 1 g of lithium chloride that dissolved in 20 mL NMP at 0°C. The whole mixture was subsequently stirred for 6–8 h. The solution was poured onto ice to give brownish to back precipitates. By filtration, the solid precipitate was separated then washed with dilute solution of sodium bicarbonate. Later, the products washed with water, ethanol, and acetone in order to remove all unreacted monomer and biproducts. Then, the product was dried at 80°C for 2 days under reduced pressure. Four new derivatives of polyamides **6_a-d_** were synthesized using the above general procedure. Table 1 shows the experimental and spectral details for the synthesis.

**Table 1.** Experimental and spectral details for polyamides **6_a-d._**

	Experimental Details	FT-IR Spectral Details
Polymer Number	amounts (g), time (h), color and yield (%)	KBr disc, υ (cm^−1^)
6_a_	The polymerization of monomer 5 (2 mmol, 0.936 g) and terephthaloyl chloride (0.002 mol, 0.406 g) for 8 h, yielded this polymer as black powder; yield 85%.	N-H of the secondary amino group 1375 cm^−1^, amide carbonyl 1670 cm^−1^, C = O cyclopentanone 1640 cm^−1^ and C = C of arylidene linkage 1610 cm^−1^, CH stretching of aliphatic 2950 cm^−1^, CH stretching of aromatic 3130 cm^−1^
6_b_	The polymerization of monomer 5 (2 mmol, 0.936 g) and isophthaloyl chloride (0.002 mol, 0.406 g) for 8 h, yielded this polymer as black powder; yield 80%.	N-H of the secondary amino group 1370 cm^−1^, amide carbonyl 1665 cm^−1^, C = O cyclopentanone 1660 cm^−1^ and C = C of arylidene linkage 1600 cm^−1^, CH stretching of aliphatic 2890 cm^−1^, CH stretching of aromatic 3150 cm^−1^
6_c_	The polymerization of monomer 5 (2 mmol, 0.936 g) and adipoyl chloride (0.002 mol, 0.336 g) for 6 h, yielded this polymer as brownish powder; yield 80%.	N-H of the secondary amino group 1340 cm^−1^, amide carbonyl 1695 cm^−1^, C = O cyclopentanone 1665 cm^−1^ and C = C of arylidene linkage 1590 cm^−1^, CH stretching of aliphatic 2920 cm^−1^, CH stretching of aromatic 3143 cm^−1^
6_d_	The polymerization of monomer 5 (2 mmol, 0.936 g) and sebacoyl chloride (0.002 mol, 0.478 g) for 7 h, yielded this polymer as brownish powder; yield 70%.	N-H of the secondary amino group 1350 cm^−1^, amide carbonyl 1690 cm^−1^, C = O cyclopentanone 1665 cm^−1^ and C = C of arylidene linkage 1585 cm^−1^, CH stretching of aliphatic 2900 cm^−1^, CH stretching of aromatic 3155 cm^−1^

### 2.4. Experimental procedures for the adsorption process

All stock standard solutions of Hg^+2^, Co^+2^, Pb^+2^, Cu^+2^, Zn^+2^, Y^+3^, Ni^+2^, and Cr^+3^ were prepared in 18.2 million ohm-cm (MΩ-cm) distilled deionized water and stored in the dark at 4°C. This value (18.2 MΩ-cm) represents the resistivity of deionized water. For the elective study, standard solutions of 2 mg/L of Hg^+2^ (or other metal ions) were prepared and individually mixed with 20 mg synthesized new polymers. Furthermore, standard solutions of 2 mg/L Hg^+2^ ion were prepared and adjusted to pH values ranging from 1.0 to 11.0 with appropriate buffer solutions. All standard solutions were individually mixed with 20 mg polymer **6_c_** in order to study the effect of pH on the selectivity of polymer 6_c_ adsorption across Hg^+2^. Mechanically shaker was applied for all mixture for 1 h at 150 rpm at room temperature. Regarding the  study of the adsorption capacity of Hg^+2^ under batch conditions, standard solutions of 2, 5, 10, 20, 50, 75, 100, 125, 175 and 200 mg/L Hg^+2^ were prepared as above procedure and adjusted to the optimum pH value of 1.0 and individually mixed with 20 mg polymer **6_c_** using a mechanical shaker.

### 2.5. Polymer characterizations and measurements

A Gallen-kamp Melting Point apparatus with a digital thermometer was used to measure the melting points for the monomers and pre-monomers. Fourier transform Infrared spectrophotometer (FT-IR) spectra were recorded on Nicolet 6700 – Thermo Fisher Scientific, by using the KBr pellet technique. ^1^H-NMR was recorded on Bruker Advance 600 MHz using CDCl_3_ and DMSO_d6_ as solvents using TMS as an internal reference. The polymer's solubility was measured using different solvents. Fifty milligrams of each polymer were mixed with 1 mL of the selected solvent. Then, the solution analyzed by visual inspection. The solubility of each powdery polymer was characterized at room temperature in excess of solvent. These solvents were dimethylsulfoxide (DMSO), dimethylformamide (DMF), n-hexane, Dichloromethane, acetonitrile, formic acid, and concentrated sulfuric acid. X-ray diffraction patterns of all polyamides were studied using Philips X-pert pro diffractometer and operated at 40 kV voltages and 40 mA current using monochromatic Cu-Kα radiation in the 2*θ* range from 5° to 80° in steps of 0.02°, with a sampling time of 1 s per step. Thermogravimetric analysis (TGA) and differential thermo-gravimetric (DTG) measurements were performed on a TA 2000 thermal analyzer. All measurements were carried out in a nitrogen atmosphere and under the same conditions with a heating rate of 10ºC/min. The mass loss was plotted against increasing temperature as well as its first derivative (DTG) that included the change in decomposition rate. Gel permeation chromatography (GPC) measurements were carried out using GPC using Agilent-GPC Agilent technologies. The refractive index detector was G-1362A with 100–104–105 A° Altrastyragel columns connected in series. THF was used as the eluent with a flow rate of 1 mL/min. Commercially available linear polymethylmethacrylate and polystyrene standards were used to calibrate the columns. The GPC apparatus was run under the following conditions: flow rate = 2.000 mL/min., injection volume = 100.000 μL, sample concentration = 1.000 g/L. The morphological features for the new polymers were examined by FE-SEM using a Jeol JSM-5400 LV instrument. The FE-SEM sample was prepared by putting a smooth part of polymer powder on a copper holder and then coating it with a gold-palladium alloy. FE-SEM images were recorded using a Penta Z Z-50P Camera with Ilford film at an accelerating voltage of 10kV using a low dose technique. A Perkin Elmer inductively coupled plasma-optical emission spectrometer (ICP-OES), model Optima 4100 DV (USA), was used for the evaluation of metal ions. The ICP-OES instrument was optimized daily before evaluation. The ICP-OES spectrometer was used at the following parameters: FR power, 1300 kW; frequency, 27.12 MHz; demountable quartz torch, Ar/Ar/Ar; plasma gas (Ar) ﬂow, 15.0 L min^−1^; auxiliary gas (Ar) ﬂow, 0.2 L min^−1^; nebulizer gas (Ar) ﬂow, 0.8 L min^−1^; nebulizer pressure, 2.4 bar; glass spray chamber according to Scott (Ryton), sample pump ﬂowrate, 1.5 mL min^−1^; integration time, 3 s; replicates, 3; wavelength range of monochromator, 165–460 nm.

## 3. Results and discussion

In this approach, we synthesize a new category of polyamides based on heteroaromatic sulfur and containing arylidene linkage in the polymer backbones. Arylidene polymers have been used in different areas of interest. These kinds of polymers have interesting features, such as highly stable thermal stability, fluorescence, photocuring capacity, good adhesion behavior, photoresists, and electronic conductivity. Variable studies on the synthesis and properties of new polymers Based on diarylidenecycloalkanones synthesis and properties, an attractive study have been reported in the literature, which included an interesting morphology, liquid-crystalline behavior, high thermal stability, corrosion inhibition, electrical conducting, antimicrobial properties, and variable properties that have been described in the literature [24–29].

### 3.1. The chemistry of polymer syntheses

As it is well known, the condensation of diamines or their derivatives with dicarboxylic acid chlorides in polar solvents such as DMF, NMP, or *N,N*’-DMAc is the basic synthesis of polyamide polymers [30]. As a continuation, our approach facilitated and investigated the synthesis and characterization of novel polymers containing arylidenecycloalkanone moieties with interesting properties. Therefore, synthesis of a new class of heteroaromatic sulfur-containing polyamides **6_a-d_** carry both types of aliphatic and aromatic species in their polymer backbones using polycondensation technique at low temperature [25, 28, 31].

**Figure 1.**
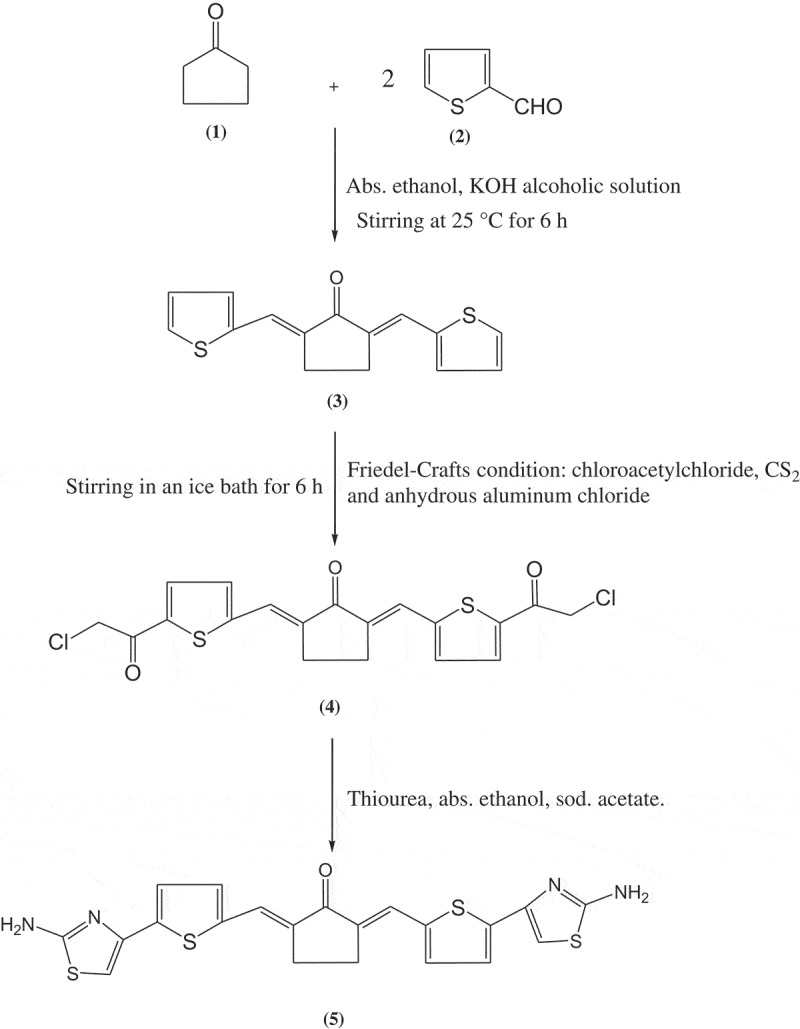
Synthetic route for new monomer **5.**

The desired polymers have been applied as highly selective adsorbents for Hg^+2^ metal ion from aqueous solution. Not only the properties of new polymers are investigated but also the synthesized new monomer and pre-monomer were determined as well. The synthesis of first pre-monomer, 2,6-bis(thiophen-2-ylmethylene)cyclopentanone **3** is reported in the literature [22, 23] by applying the condensation of 10 mmol of cyclopentanone with 22 mmol of 2-thiophencarbaldehyde in absolute ethanol and potassium hydroxide solution as a basic catalyst. The prepared pre-monomer had been easily converted to 2,5-bis[4-choloroacetyl(thiophen-2-ylmethylene)]cyclopentanone pre-monomer **4** according to the well-known Friedel-Crafts condensation method. This reaction is indicated the interaction of pre-monomer **1** with chloroacetylchloride in the presence of carbon disulfide and anhydrous aluminum chloride. Our new monomer **5** was synthesized by the interaction of pre-monomer **4** with thiourea and in the presence of sodium acetate as illustrated in (Figure 1). The chemical structures of all pre-monomers and new monomer **5** were confirmed by spectral and elemental analysis and illustrated the melting points, FT-IR and ^1^H NMR, spectra which supported the proposed structures as represented in the experimental part.

**Figure 2.**
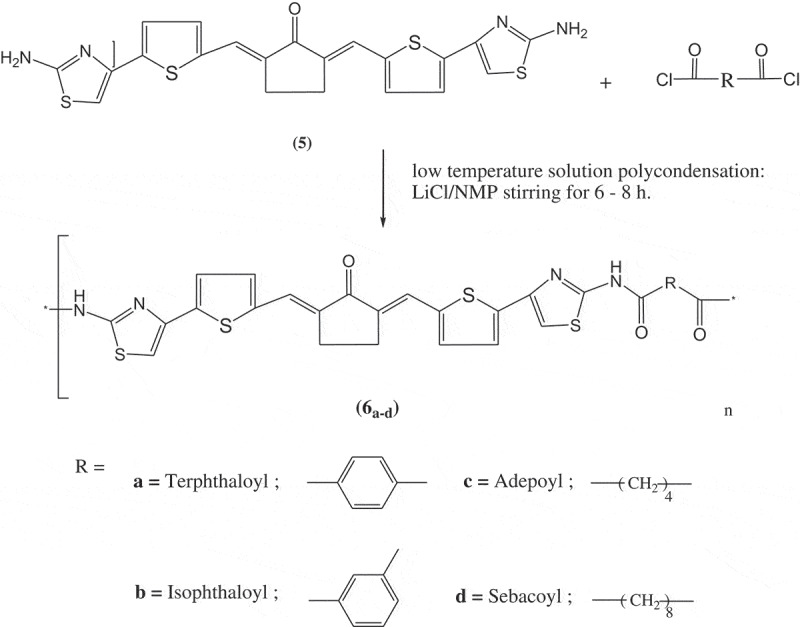
Synthesis of polyamides **6_a-d._**

Four derivatives of heteroaromatic sulfur-containing polyamides **6_a-d_** and containing arylidene linkage have been synthesized by the reaction of monomer **5** with two aliphatic and two aromatic diacid chlorides using low-temperature polycondensation technique as given in Figure 2. The chemical structures of these new polymers were confirmed by FT-IR, which analyses were scanned from 4000 to 400 cm^−1^. Table 1 represents the significant FT-IR peaks for these polyamides. New characteristic absorption bands were attributed to N-H of the secondary amino group in the range 3340–3375 cm^−1^, amide carbonyl bands at 1665–1695 cm^−1^, beside the basic characteristics absorption bands that present in the polymer main chains such as, carbonyl of the cyclopentanone moiety at nearly 1640–1665 cm^−1^ and C=C bond of arylidene bands at 1590–1620 cm^−1^.

### 3.2. Polymers' identifications and characterizations

The synthesized polymers were inspected with different characterization techniques, including solubility, GPC molecular weight determinations, XRD diffraction patterns, thermal behavior, and SEM morphological features.

The solubility properties of the novel polymers **6_a-e_** are measured at room temperature using six different solvents as chosen examples. The test procedure was described in the experimental part as reported in the literature and the solubility results are shown in Table 2 [23–25,32]. From those results, it is noticeable that all polymers are clearly soluble in both formic and concentrated sulfuric acids, which represent good examples for protonic solvents. On the contrary, all derivatives show bad solubility behavior in methylene chloride and n-hexane as examples for non-polar solvents. All polymers were completely insoluble in these solvents. While in DMSO and DMF as examples for polar aprotic solvents, polymers **6_c,d_** with aliphatic spacers show completely solubility behavior, whereas, polymers **6_a,d_** with aromatic spacers are partially soluble in both solvents. A similar solubility behavior has been observed compared to other cyclopentanone containing the polymer. This observation is due to the presence of heteroaromatic sulfur in the form of thiopheno- and thiazolo-species, which are linked to the polymer's main chains [27, 28]. Although, a lower solubility behavior has been reported for these polymers that other reported polymers, which contain cyclohexanone moieties due to the presence of cyclopentanone moiety [26, 28, 33]. Furthermore, there is no difference in the solubility behavior between polymers based on aliphatic spacers (CH_2_)_4_ and (CH_2_)_8_, where both polymers have similar solubility behavior in all selected solvents.

**Table 2.** Room temperature solubility character for polyamides **6_a-d._**

Polymer Number	DMSO	DMF	n-Hexane	CH_2_Cl_2_	HCOOH	H_2_SO_4_
6_a_ 6_b_ 6_c_ 6_d_	•° •° •• ••	•° •° •• ••	°° °° °° °°	°° °° °° °°	•• •• •• ••	•• •• •• ••

••: Soluble at room temperature.

•°: Partially soluble.

°°: Insoluble.

The molecular weights of polyamides **6_c_** and **6_d_** are measured by GPC using Agilent-GPC Agilent technologies and the experiment details are described in the experimental part. The molecular weight of polymers **6_a_** and **6_b_** by GPC is not available due to the solubility factor. These molecular weight values were calculated by means of a computer program. Table 3 presents the results for GPC for both polymers **6_c_** and **6_d_**. From the data in Table 3, the tested polymers had nearly the same chain length. This outcome was confirmed from the average molecular weight (*M_w_*) results. The *M_w_* values for polymers **6_c_** and **6_d_** are 50,127.4 and 58,965.2, respectively. While number-average molecular weights (*M_n_*) of the same polymers are 45,563.2 and 54,652.9. In addition, polymer **6_c_** provides an average number of repeating units (*P_w_*) = ~87 and its PDI = 1.10. Whereas, polymer **6_d_** has an average number of repeating units (*P_w_*) = ~93 and it’s PDI = 1.08.

**Table 3.** GPC molecular weight results of polyamides **6_a-d._**

	^a^GPC			
Polymer Number	^b^*M_w_*	^c^*M_n_*	^d^*P_w_*	PDI
6_a_ 6_b_ 6_c_ 6_d_	n/a n/a 50127.4 58965.2	n/a n/a 45563.2 54652.9	n/a n/a ~ 87 ~ 93	n/a n/a 1.10 1.08

^a^GPC measurements were carried out in THF. ^b^Weight-average molecular weight. ^c^number-average molecular weight ^d^Average number of repeating units

**Figure 3.**
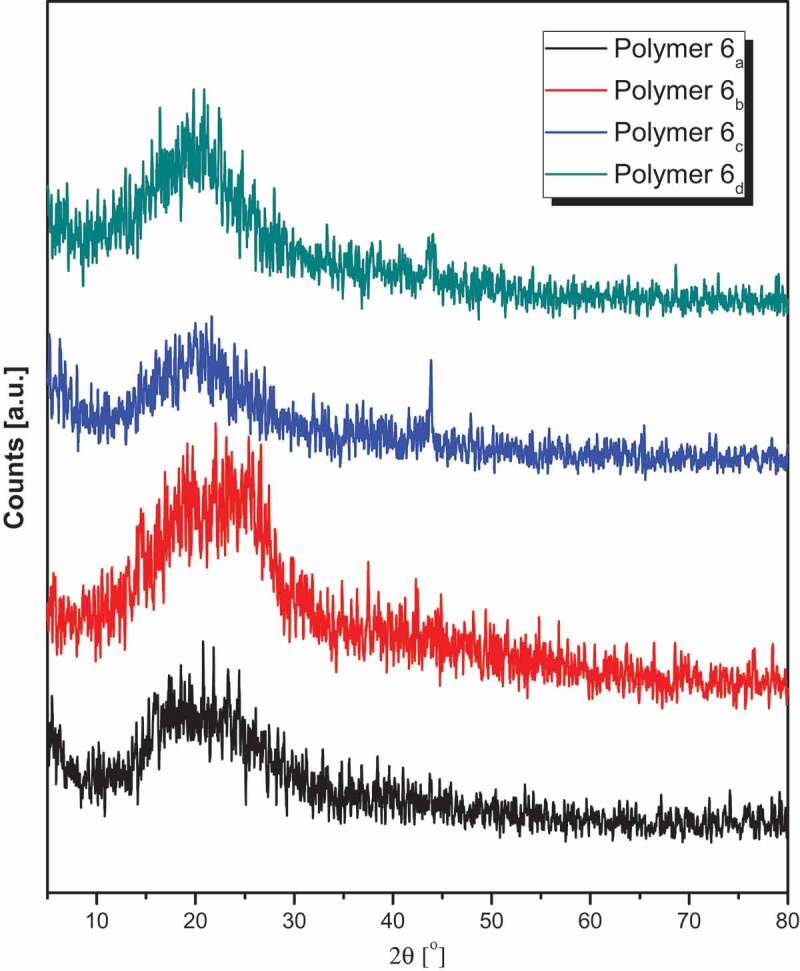
XRD diffraction patterns of polyamides **6_a-d._**

The X-ray diffraction battens for all synthesized polyamides **6_a-d_** are examined by Philips X-pert pro diffractometer over the 2*θ* region = 5–80° as illustrated in Figure 3. Polymers **6_a_** and **6_b_** are completely amorphous in nature and show a two overlapped halo peaks in the 2*θ* range 13–20° and 21.5–31.8°. Both polymers have no degree of crystallinity with no order of crystalline structures. Although polymers **6_c_** and **6_d_** still have the same amorphous nature of previous polymers, they show a small peak at 2*θ* value 43.83°, in addition the wider broad peaks in the 2*θ* range 13.27–27.8°. Hence, it can be classified as semi-crystalline behavior as it shows some diffraction peaks that are intermediate between crystalline and amorphous interference. This proved a class of structures that had an intermediate ordered state between crystals in their atoms and molecules arrangement. The small increase in the crystallinity behavior of both polymers might attribute to the enhanced in the polymer chain flexibility and that may be responsible for the attractions of adjacent chains [34]. Comparing these polymers with other published polymers based on cyclohexanone moieties [27, 28], it is clearly mentioned that the cyclopentanone moiety effectively decreases the crystallinity of the polymers and this is mainly due to the extent of flexibility of cyclohexanone moiety compared to the considerable rigidity of cyclopentanone moiety [35].

**Figure 4.**
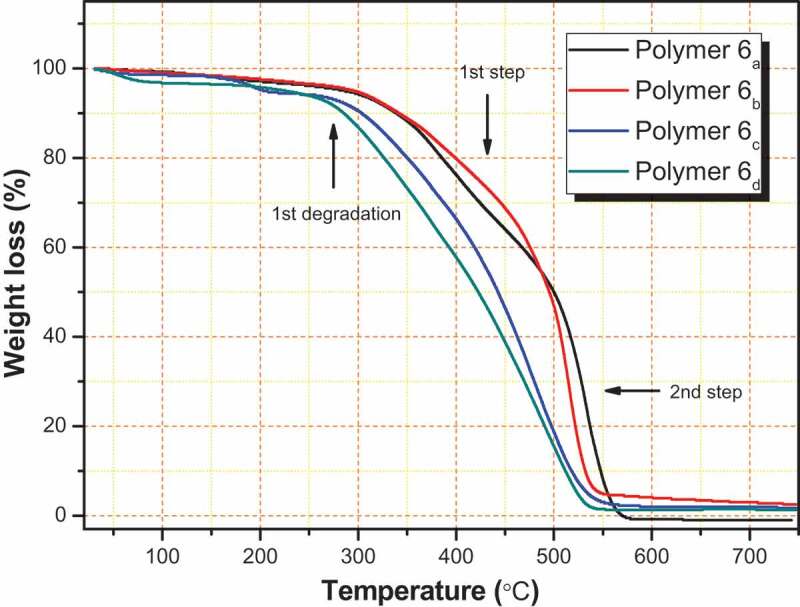
TG curves of polyamides **6_a-d_** under nitrogen at a heating rate of 10°C/min

**Figure 5.**
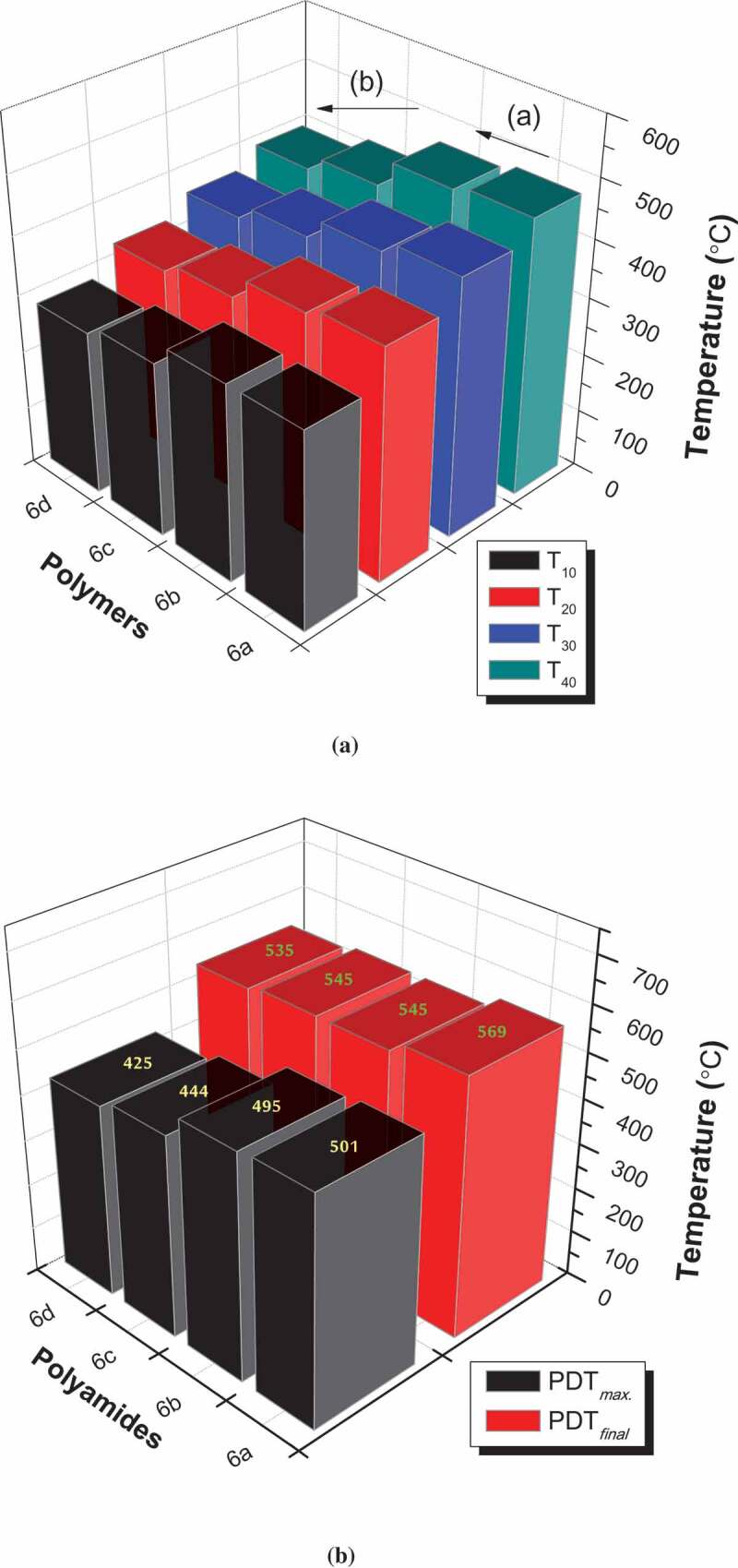
a: Temperature (°C) for various percentage decompositions (T_10_, T_20_, T_30_, and T_40_) of polyamides **6_a-d_** b: PDT*_max._* and PDT*_final_* values for polyamides **6_a-d._**

The thermal behavior of our targeted sulfur-containing polyamides **6_a-d_** is tested by TGA and DTG under a nitrogen atmosphere with a heating rate of 10ºC/min as shown in Figure 4 and Table 4. The TG curves of all polymers show a small weight-loss in the range 1–3%, which might be due to loss of entrapped solvents and moisture. In addition, they had a similar pattern of degradation. The decomposition for all derivatives is liable to the nature of these polymers and occurs mainly in two overlapped steps. Only polymer 6a shows considerable separation between both degradation steps. The first degradation step starts at 271°C and ends at 460°C; while the second step stars at 465°C and ends at 570°C. Whereas the other three derivatives show a clear overlapped degradation steps in the range of 275–540°C which cannot be easily separated by the normal view. The decomposition was included pyrolytic oxidations of C C double bonds that separated from many bonds with the liberation of free shorter chains depending upon the nature of these polymers and formation of char as end products [24, 25, 36]. The solid residue left (char end products) at 600°C is illustrated as R_600_. R_600_ values for all the synthesized polymers are in the range of 0.5–4%. T_10_ – T_40_ values indicated the temperatures for various % weight losses at 10, 20, 30 and 40 weight losses percentage as shown in Table 4 and Figure 5(a). The initial decomposition of new polymers does not occur before T_10_ values. Therefore, it was considered as polymer-decomposition temperature (PDT) [23 37,]. PDT was tested in the range 283–337°C. The degradation step in all derivatives did not start before ~280°C and this due to claim that these polymers are thermally stable at high temperature. Also, all polymers show good thermal stability behavior due to they have high temperatures at T_10_. The order of higher PDT values is **6**_a_ > **6**_b_ > **6**_c_, **6**_d_. The higher polymer degradation temperature (PDT*_max_*) was due to the temperature value at which the maximum rate of weight loss occurred [38–39–40]. PDT*_max_* for all the polymers in the range of 425°C–501 ºC are stated in Table 4. The final polymer degradation temperature (PDT*_final_*) related to the temperature at which the rate of degradation that may occur is nearly completed [23, 41–43]. The TG curves show that, the PDT*_final_* for all polymers, were nearly completed in the range 535 °C – 569°C. The order of higher thermal stability at PDT*_max_* and PDT*_final_* is completely the same **6**_a_ > **6**_b_ > **6**_c_, **6**_d_ as illustrated in Figure 5(b). Polymers **6_c,d_** show lower thermal stability than those polymers **6_a,d_** at all giving temperatures for various weight losses percentage. This observation is almost attributed to the higher flexibility of both polymers due to the presence of (CH_2_)_4_ and (CH_2_)_8_ as aliphatic spacers. Such behavior is easily detected at all temperatures for various weight losses percentage T_10_ – T_40_ [23, 24, 41]. In addition, polyamide 6_d_ contained longer aliphatic chain (eight carbons) shows the lowest thermal stability behavior in all the synthesized polymers.

**Table 4.** Thermal properties of polyamides **6_a-d._**

	Temperature (°C) for Various Percentage Decompositions ^a^					
Polymer Number	T_10_	T_20_	T_30_	T_40_	PDT*_max_*^b^ (°C)	PDT*_final_* ^a^ (°C)
6_a_	337	397	442	476	501	569
6_b_	337	384	442	466	495	545
6_c_	300	345	386	418	444	545
6_d_	283	327	361	392	425	535

^a^The values were determined by TGA at heating rate of 10°C min^−1^ under nitrogen atmosphere

^b^Determined from DrTGA curves.

**Figure 6.**
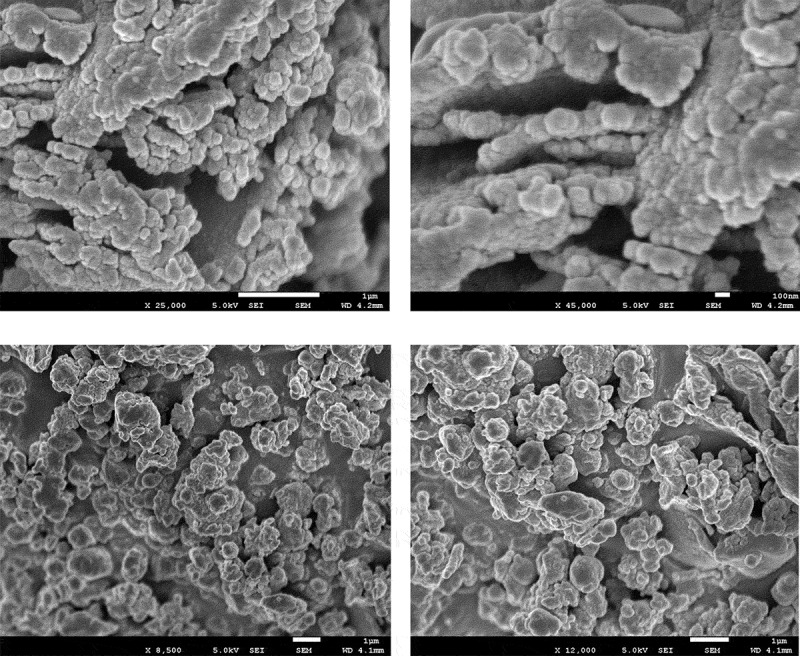
FE-SEM micrographs of polymers **6_a_** (a: x = 25,000, b: x = 45,000) and **6_c_** (c: x = 8500, d: x = 12,000)

The morphological studies of polymers **6_a_** and **6_b_** as chosen examples are measured by FE-SEM measurements using a Jeol JSM-5400 LV instrument to investigate the polymer's morphology. FE-SEM micrographs are taken using a Penta Z Z-50P camera with Ilford film at an accelerating voltage of 10kV using a low dose technique [44]. FE-SEM images illustrated both polymers surfaces are nearly similar. The surface of polymer **6_a_** shows that it consists of merged globular particles in both higher and lower magnifications. The particles are aggregated to produce bigger grains with a considerable void. The average diameters of each particle are nearly about 100 nm as represented in Figure 6,_a,b_ with magnifications X = 25,000 and X = 45,000, respectively. While the surface of polymer **6_b_** shows completely fussed accumulated globular grains which form a rock-like structure as represented in Figure 6_c_,_d_ with magnifications X = 8,500 and X = 12,000, respectively.

### 3.3. Metal uptake and surface selectivity study toward Hg^+2^ ion

There is no doubt that the wastewater treatments become recently more feasible from the economical point of view with special attention to its high impact in the industries. After all the challenge to produce efficient adsorbents, a lot of efforts have been reported in variable fields of industries through the developments of solid adsorbents from variable resources which have been utilized in the wastewaters treatment. The synthesized polymers were considered as one important source of these products. Therefore, polymers **6_a-d_** are used as surface selective adsorbents to extract toxic Hg^+2^ ions from aqueous solution. All synthesized products were tested as adsorbent and yielded significant adsorption efficiency while tested in a preliminary test. More particularly, the polymer **6_c_** represents the highest adsorption efficiency among all four polymers; thus, it is selected as a surface selectivity study example in this approach. It has been provided that there is a promising synergetic effect which enhances the absorption process of Hg^2+^ than the other metal ions by our new heteroaromatic sulfur-containing polymers. This is due to the presence of thiophene moieties (which contains S & N atoms) in the polymers' main chains. Together with the secondary amino linked groups that present through the formation of these new polyamides; which can all consider as protonated centers at lower pH values. On the other hand, Hg^+2^ can be presented in different forms at lower pH values including: HgCl_2_, HgCl_3_^−1^ and HgCl_4_^−2^ as neutral or chloroanionic complexes respectively. Therefore, these chloroanionic complexes can easily be stuck over the protonated centers in the polymer backbones which formed at lower pH values through electrostatic attraction forces. Hence, only Hg^+2^ can be absorbed via these new polymers.

The desorption capacity and other common optimization steps were established in more details. The distribution coefficient selectivity of polymer **6_c_** across different metal ions was obtained. The distribution coefficient (*K_d_*) is calculated using the following equation:

(1) $$K_{d}=\frac{\left(C_{0}-C_{e}\right)}{C_{e}}\times \frac{V}{m}$$

where *C_o_* and *C_e_* are the initial and ﬁnal concentrations before and after ﬁltration with the adsorbent, respectively, *V* is the volume (mL) and *m* is the weight of the adsorbent (g). For all metal ions, the distribution coefficient values are investigated in this study are illustrated in Table 5.

**Table 5.** Selectivity study on the adsorption of polymer **6_c_** toward different metal ions at 20°C

Metal Ion	*q_e_* (mg g^−1^)	*K_d_* (mL g^−1^)
Hg^+2^	1.95	42,196.54
Co^+2^	0.93	869.16
Pb^+2^	0.51	337.79
Cu^+2^	0.39	239.16
Zn^+2^	0.36	221.75
Y^+3^	0.14	75.27
Ni^+2^	0.10	52.08
Cr^+3^	0.07	37.34

**Figure 7.**
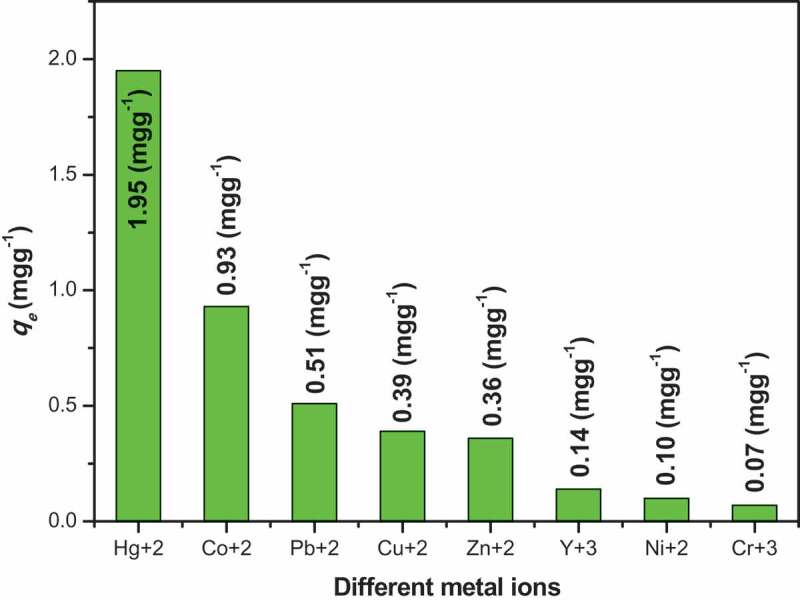
Selectivity study of polymer 6_c_ toward different metal ions

As shown in Table 5, polymer **6_c_** represents the higher distribution coefficient value (42,196.54 mL g^−1^) across Hg^+2^ among all the tested metal ions. These resulted data illustrate the selectivity of the synthesized polymer derivatives across Hg^+2^ is most likely preferably compared to other metal ions that tested in this study as illustrated in Figure 7.

#### 3.3.1. Effect of pH

**Figure 8.**
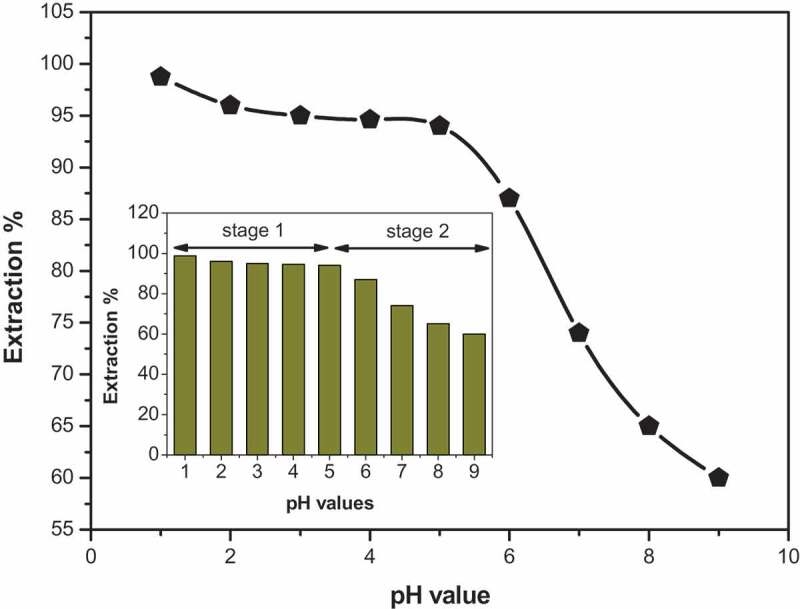
The effect of pH on the adsorption of 2 mg/L Hg^+2^ on 20 mg polymer **6_c_** at 20°C

One of the most valuable optimization tools on the adsorption process is studying the pH effect of new polyamides. The presence of H^+^ ions in the solution has three main roles, which are the metal ions removal from aqueous media by adsorption, which affects the degree of ionization and species of adsorbate [45]. Therefore, the effect of pH on the Hg^+2^ adsorbed by the new polymer **6_c_** is investigated and illustrated in Figure 8. A concentration of nearly about 2 mg/L Hg^+2^ is used, and the effect of pH on the reaction is monitoring by varying the solution pH range from 1.0 to 9.0 with corresponding buffer solutions. All the standard solutions are separately mixed with 20 mg of polymer **6_c_**. Figure 8 represents the effect of solution pH on the extraction percentages. As it is reported in Figure 8, pH values have a direct effect on the adsorption process. The test was indicated using 20 mg of polymer **6_c_** at 20°C and under a range of pH values from 1.0 to 9.0. The results in Figure 8 also represent an increase followed by a subsequent decrease in the extraction percentage of Hg^+2^ with increasing pH values. The pH effect is classified into two main steps. The first stage shows a gradual decrease from pH 1 to pH 5; while the second stage is ranged from pH 5 to pH 9 and shows a considerable decrease. Hence, it is easy to say that the pH impact has clearly affected the adsorption process electively in the stage 2 rather than stage 1.

The highest extraction percentage of Hg^+2^ is reached (98.75) at pH 1.0, which shows that the polymer **6_c_** is most selective across Hg^+2^ at previous pH value. The reason of the highest percentage of Hg^+2^ extraction and selectivity with polymer **6_c_** at this pH value is explained by the electrostatic interaction which may occur between secondary amino groups of the selected polymer or the protonated sites, presented on carbonyl groups, at pH 1.0, and the negatively charged species (HgCl_4_^−2^, the primary form of Hg^+2^ in HCl solution) together with the neutral HgCl_2_ and other chloroanionic complex HgCl_3_^−1^. Therefore, Hg^+2^ is highly removed from the matrix. From the results, the pH value of 1.0 is chosen for the study of other parameters controlling the maximum uptake on the selected polymer **6_c_** under static conditions.

#### 3.3.2. Adsorption capacity detection

**Figure 9.**
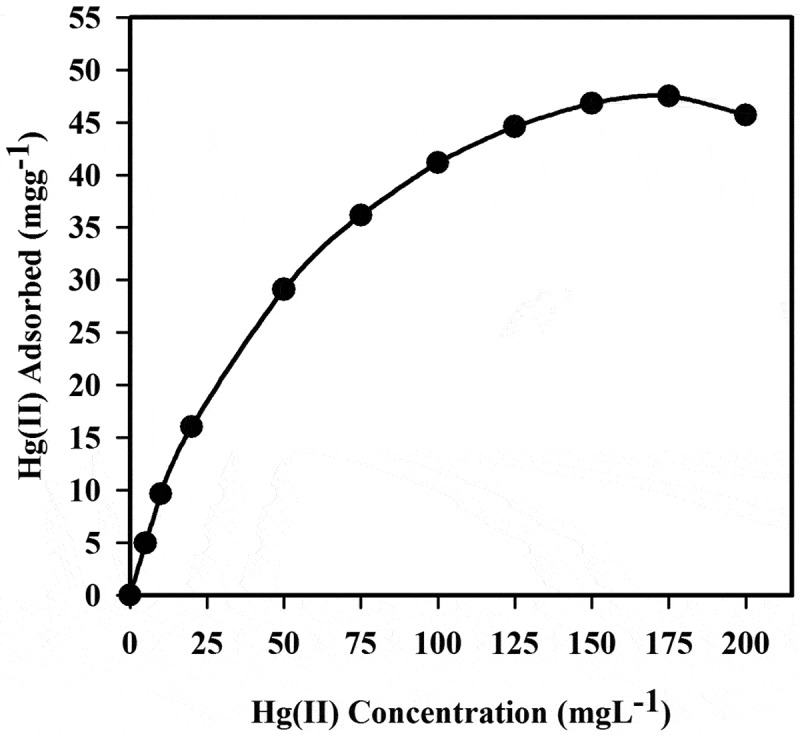
Adsorption proﬁle of Hg^+2^ on 20 mg polymer **6_c_** in relation to the concentration at pH 1.0 and 20°C

The adsorption capacity of Hg^+2^ is determined by using different amounts of Hg^+2^ separately mixed with 20 mg polymer 6_c_ at pH 1.0 under a batch procedure. Standard solutions of 2, 5, 10, 20, 50, 75, 100, 125, 175 and 200 mg/L Hg^+2^ were previously prepared before attempting the required determination procedures. The adsorption isotherm study, the adsorption capacity of polymer 6_c_ for Hg^+2^ is found to be 47.95 mg g^−1^ by adsorption isotherm study, as shown in Figure 9. This is significantly higher than the previously reported adsorption capacity for Hg^+2^ that use different solid adsorbents [41–52], as illustrated in Table 6.

**Table 6.** Selected adsorption capacity studies against Hg^+2^ using different adsorbents

Solid Adsorbent	Adsorption Condition Temp. (°C)/pH	q_m_ mg g^−1^	References
Procion brown MX 5BR immobilized pHEMA/chitosan)	20 °C/pH = 5	68.20	[46]
Chitosan immobilized reactive yellow 2 dye	20 °C/pH = 5.5	39.60	[47]
Procion Green H-4G immobilized pHEMA/chitosan	20 °C/pH = 5.5	48.10	[48]
Mesoporous carbon MC	20 °C/pH = 6-7	7.4	[49]
AEPE-monotmorillonite	25 °C/pH = 4	46.1	[50]
Carbons SHC	30 ± 2 °C/pH = 5.5	43.86	[51]
MnO2/CNT nanocomposite	25 °C/pH = 6	35.69	[52]
Heteroaromatic sulfur containing polyamides	20 °C/pH = 1	47.95	This work

In Figure 9, the adsorption capacity is progressively increased with increasing the metal ion concentration tell the critical saturation point. However, a minimal decrease in the adsorption capacity of the polymer 6_c_ for Hg^+2^ after saturation has been observed. Also, the highest concentration of Hg^+2^ 175 mg/L is obtained due to the saturation of the binding sites of polymer 6_c_ with HgCl_4_^–^ species. Therefore, an insignificant impact of concentration might be noted in the maximum uptake capacity of polymer 6_c_ for Hg^+2^ after this saturation process. The stability of the selected polymer has been determined over three cycles, resulting closely with the same adsorption capacity due to its significant stability property that can reuse with complete efficiency.

#### 3.3.3. Adsorption isotherm models

**Figure 10.**
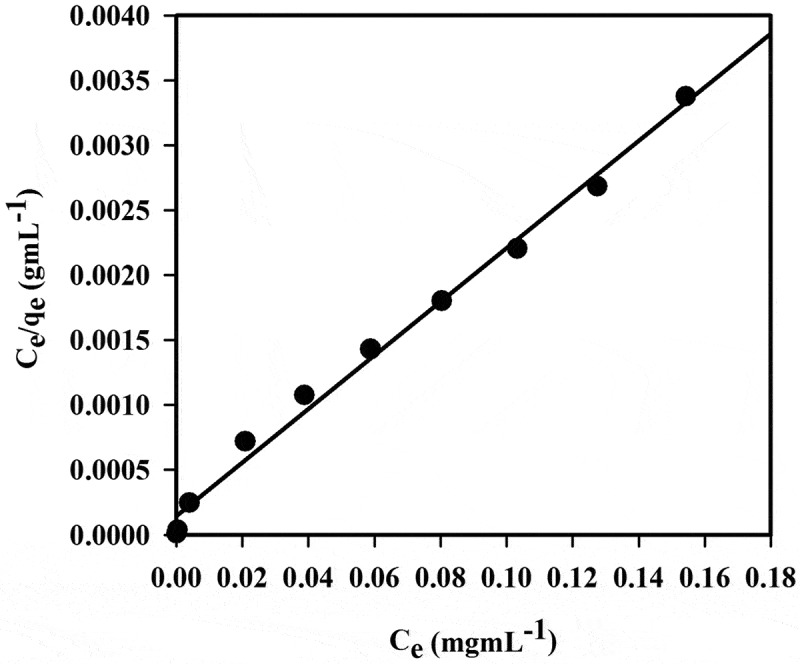
Langmuir adsorption isotherm model of Hg^+2^ adsorption on 20 mg polymer **6_c_** at pH 1.0 and 20°C. Adsorption experiments were obtained at different concentrations (1–200 mg/L) of Hg^+2^ under batch conditions

Applying adsorption isotherm models in this study is ideal to analyze the results. Both Langmuir and Freundlich adsorption models were used to illustrate the polymer isotherms [53, 54]. Experimental data are fitted well by the Langmuir equation, as presented in Figure 10.

The Langmuir isotherm model is used to measure the uniformity of non-interacting surface area. The Langmuir classical adsorption isotherm is illustrated as follows [55]:

(2) $$C_{e}/q_{e}=\left(C_{e}/Q_{e}\right)+1/Q_{o}b$$

where *C*_e_ is the concentration of metal ion in solution at equilibrium (mg mL^−1^) and *qe* refers to the amount of metal ion per gram of the adsorbent at equilibrium (mg g^−1^). The symbols *Q_o_* and b are the Langmuir constants for polymer 6_c_ and are related to the maximum Hg^+2^ adsorption capacity (mg g^−1^) and affinity parameter (L mg^−1^), respectively. From the linear plot of *C_e_/q_e_* against *Ce*, Langmuir constants *Q_o_* and b could be calculated with a slope and intercept equal to 1/*Q_o_* and *1/Q_o_b*, respectively. In addition, the important characteristics of the Langmuir adsorption isotherm model can be calculated in terms of a dimensionless constant separation factor or equilibrium parameter, *R_L_*, which is deﬁned as follows:

(3) $$R_{L}=1/(1+bC_{o})$$

where *b* is the Langmuir constant that indicates the adsorption nature and different isotherm shapes, and *Co* refers to the initial concentration of Hg^+2^. The value of *R_L_* represents the adsorption isotherm nature, and values between 0< *R_L_* < represent favorable adsorption [56]. Based on the least-squares ﬁt, a linear plot is obtained from the Langmuir isotherm equation, which proves the validity of the Langmuir adsorption isotherm model for the adsorption process as given in Figure 10. By testing the above results, it appears that the homogeneous surface for polymer 6_c_ is a monolayer during the adsorption process. Table 7 illustrates the Langmuir isotherm constants for adsorption of Hg^+2^ on polymer 6_c_ surface. Langmuir constants were calculated for *Q_o_* and *b* as 48.41 mg g^−1^ and 0.14 L mg^−1^, respectively. Regarding Langmuir model, the correlation coefficient (*R^2^*) was 0.993 for adsorption of Hg^+2^ on the surface of selected polymer 6_c_, denoting that the data is ﬁtted with the Langmuir model. The *R_L_* value of Hg^+2^ adsorption on polymer 6_c_ surface is 0.04, which supports a highly favorable adsorption process of polymer 6_c_ based on the Langmuir model. From the Langmuir equation, the calculated Hg^+2^ adsorption capacity is (48.41 mg g^−1^), which is in good agreement with that experimentally obtained from the adsorption isotherm study (47. 5 mg g^−1^).

**Table 7.** Parameters of Langmuir isotherm constants for adsorption of Hg^+2^ polymer 6c surface, at pH 1.0 and 20°C (*N *= 3)

Polymer Number	*Q*o (mg/g)	*b* (L mg^− 1^)	*R2*	*RL*
6_c_	48.41	0.14	0.993	0.04

## 4. Conclusions

Four novel heteroaromatic sulfur-containing polyamides **6_a-d_** are synthesized using polycondensation technique at low temperature. The synthesized polymers have been tested as surface selective adsorbents for the removal of toxic Hg^+2^ metal ions from aqueous solution. The structure of these new polymers, as well as monomer, is confirmed by spectral and elemental analysis giving fully assignable spectra, which are in accordance with the proposed structures. Both spectral and elemental analyses were investigated besides their solubility.

In addition, ICP-OES measurements have been exploited as an important tool for the study of the surface selectivity of polyamides 6_a-d_ across different metal ions. These ions include Hg^+2^, Co^+2^, Pb^+2^, Cu^+2^, Zn^+2^, Y^+3^, Ni^+2^, and Cr^+3^ as a wide selection from the most common metal ions. Polymer 6_c_ represents the highest adsorption efficiency, with the maximum quantity adsorbed at pH = 1. The adsorption capacity of polymer 6_c_ for Hg^+2^ is 47.95 mg g^−1^, and its adsorption isotherm is in agreement with the Langmuir adsorption isotherm. This material, high adsorption capacity, will open the field for more evolution and development of adsorbent with simple and inexpensive methods associated with high metal removal efficiency.
